# Subsequent Cancer Prevention and Control Activities Among Low- and Middle-Income Country Participants in the US National Cancer Institute’s Summer Curriculum in Cancer Prevention

**DOI:** 10.1200/JGO.19.00231

**Published:** 2019-10-18

**Authors:** Amanda L. Vogel, Camille Morgan, Kalina Duncan, Makeda J. Williams

**Affiliations:** ^1^ Clinical Monitoring Research Program Directorate, Frederick National Laboratory for Cancer Research Sponsored by the National Cancer Institute, Frederick, MD; ^2^ Center for Global Health, National Cancer Institute, National Institutes of Health, Bethesda, MD; ^3^ Center for Global Mental Health Research, National Institute of Mental Health, National Institutes of Health, Bethesda, MD

## Abstract

**PURPOSE:**

A dramatic shift in the burden of cancer from high-income countries to low- and middle-income countries (LMICs) is predicted to occur over the next few decades. An effective response requires a range of approaches to capacity building in cancer prevention and control in LMICs, including training of cancer prevention and control professionals. Toward this end, the US National Cancer Institute includes LMIC-based participants in its Summer Curriculum in Cancer Prevention, which is an annual, short-term in-person training program.

**METHODS:**

In 2015 and 2016, the US National Cancer Institute fielded a survey to all Summer Curriculum alumni who were based in LMICs when they participated in the program, between 1998 and 2015. Its aims were to learn about subsequent engagement in cancer prevention and control in LMICs and attribution of activities/accomplishments to participation in the Summer Curriculum in Cancer Prevention.

**RESULTS:**

Respondents (N = 138) worked in academia/research (n = 61), health care (n = 41), and health policy/Ministries of Health (n = 36) in all six world regions. Most respondents (90.6%) worked in the same LMIC as when they participated in the Summer Curriculum in Cancer Prevention. When asked about activities/accomplishments completed as a result of participation, 92.8% reported at least one cancer prevention and control practice activity/accomplishment, 81.2% reported at least one cancer research activity/accomplishment, and 44.2% reported authoring one or more peer-reviewed publications. Reported ways that the Summer Curriculum in Cancer Prevention contributed to these activities/accomplishments were emphasizing a public health approach; focusing on research priorities, methods, and scientific writing; and highlighting the importance of research and publications. Finally, 79.7% of respondents reported using Summer Curriculum in Cancer Prevention materials to train others.

**CONCLUSION:**

These findings have implications for the design of future training initiatives for LMIC-based cancer prevention and control professionals.

## INTRODUCTION

A dramatic shift in the burden of cancer from high-income countries (HICs) to low- and middle-income countries (LMICs) is predicted to occur over the next few decades.^1^ Contributing factors include enhanced access to cancer screening and treatment in HICs and the combined effects of increasing prevalence of cancer risk factors, better control of infectious disease, and population growth and aging in LMICs.^1,2^ Many LMICs have extremely limited capacity to address this challenge, including little infrastructure for cancer prevention, detection, diagnosis, and treatment; lack of cancer registries; limited training opportunities; and little funding for all of the above.^2-4^ An effective response requires a range of approaches to capacity building for cancer prevention and control in LMICs, including but not limited to national cancer control planning, infrastructure building, financing and procurement of medications, cancer surveillance and research, and training of cancer prevention and control professionals.^2,5^

Aligned with its mission to advance cancer research, the National Cancer Institute (NCI) of the US National Institutes of Health includes LMIC-based participants in one of its hallmark cancer training programs, the NCI Summer Curriculum in Cancer Prevention (hereafter referred to as the “Summer Curriculum”). Offered annually since 1986, the Summer Curriculum includes a 4-week course on Principles and Practices of Cancer Prevention and Control (hereafter referred to as the Principles course) and a 1-week course on Molecular Prevention. Participants may take one or both courses. It is designed for health care providers, cancer researchers, cancer prevention and control professionals, fellows, and students from the United States and abroad.^6,7^ Approximately 80 to 120 individuals participate each year.

**CONTEXT**

**Key Objective**
To provide state-of-the-art training in cancer prevention and control to participants based in low- and middle-income countries (LMICs) via the US National Cancer Institute’s Summer Curriculum in Cancer Prevention, a short-term, intensive training program.
**Knowledge Generated**
A survey of LMIC-based participants in the Summer Curriculum in Cancer Prevention documented benefits for subsequent cancer prevention and control and cancer research activities/accomplishments, peer-reviewed publications, and training others. In addition, most participants remained in the same LMIC as when they participated in the Summer Curriculum.
**Relevance**
Findings provide evidence that this short-term training program enhanced capacity in cancer prevention and control and cancer research among participants, contributed to training of others in LMICs, and did not result in brain drain. Findings may help to inform content of future cancer prevention and control training programs for LMIC-based participants. They also point to demand for expanded training opportunities and potential solutions such as train-the-trainer approaches.

LMIC-based participants represent a growing proportion of students in the Summer Curriculum. In the first decade after the Summer Curriculum opened to international participants (1998 to 2007), they comprised approximately 10% of participants; in the second decade (2008 to 2018), they comprised approximately one-third of participants. In a 2009 survey of Summer Curriculum alumni who were based in LMICs when they participated in the program, almost all respondents reported that participation enhanced their knowledge and skills (93%) and prepared them for effective contributions in their home countries (98%).^8^

In 2015 and 2016, in light of the increase in LMIC-based participants since the prior survey, the NCI Center for Global Health fielded another survey to Summer Curriculum alumni who were based in LMICs when they participated in the program. Its aims were to learn about their subsequent cancer prevention and control activities and accomplishments and the extent to which respondents attributed these to participation in the Summer Curriculum. This article reports findings and discusses implications for the design of future training initiatives for LMIC-based cancer prevention and control professionals.

## METHODS

The Summer Curriculum provides a multidisciplinary overview of principles and practices for cancer prevention and control. The faculty includes more than 50 experts from the NCI and other US research institutions. Internationally focused lectures include content relevant to the global burden of cancer, and the Principles course includes region-specific interest group meetings where participants and NCI scientists discuss their work in a region of the world, laying the groundwork for future collaborations. International applicants are evaluated for acceptance into the course on the basis of strength of interest/work experience relevant to cancer prevention and control, likelihood to use the knowledge gained, geographic balance, and academic background. There is no cost to apply or participate, and applicants from LMICs are considered for financial assistance.^7^

As a whole, evaluations of training courses tend to examine four levels of outcomes: (1) immediate satisfaction with the training, (2) knowledge acquisition, (3) application of training content to one’s work, and (4) results of having applied training content (cf, Kirkpatrick and Kirkpatrick^9^). The 2015 to 2016 survey assessed outcomes of participation in the Summer Curriculum, with a focus on levels 3 and 4. It aimed to learn about respondents’ subsequent engagement in a range of cancer prevention and control activities (level 3), and related accomplishments in cancer prevention and control (level 4), the extent to which respondents attributed these activities and accomplishments to their participation in the Summer Curriculum, and whether they continued to work in LMICs.

An invitation to participate was sent via e-mail to all 427 individuals who participated in the Summer Curriculum between 1998 and 2015 and were based in LMICs when they participated. E-mail addresses were obtained from Summer Curriculum applications. The invitation e-mail included an embedded link to the self-administered online survey. A variation of the Dillman method was used for follow-up, with reminder e-mails sent at 2 and 3 weeks after the initial invitation.^10^ Responses were anonymous. The survey was approved by the US Office of Management and Budget.

The 31-question survey included open- and closed-ended questions on years since participation; geographic and organizational location of current work and whether these changed since participation in the Summer Curriculum; completion of 18 specific activities/accomplishments in cancer prevention and control practice and research, and attribution to participation in the Summer Curriculum; subsequent peer-reviewed publications on cancer control or cancer research, and attribution to participation; and use of Summer Curriculum learning objectives and content to train others. Survey questions were informed by the prior survey of Summer Curriculum participants based in LMICs,^8^ Summer Curriculum learning objectives, topics of interest to the NCI Center for Global Health, and a review of related literature. Key survey questions are summarized in Table 1.

**TABLE 1:**
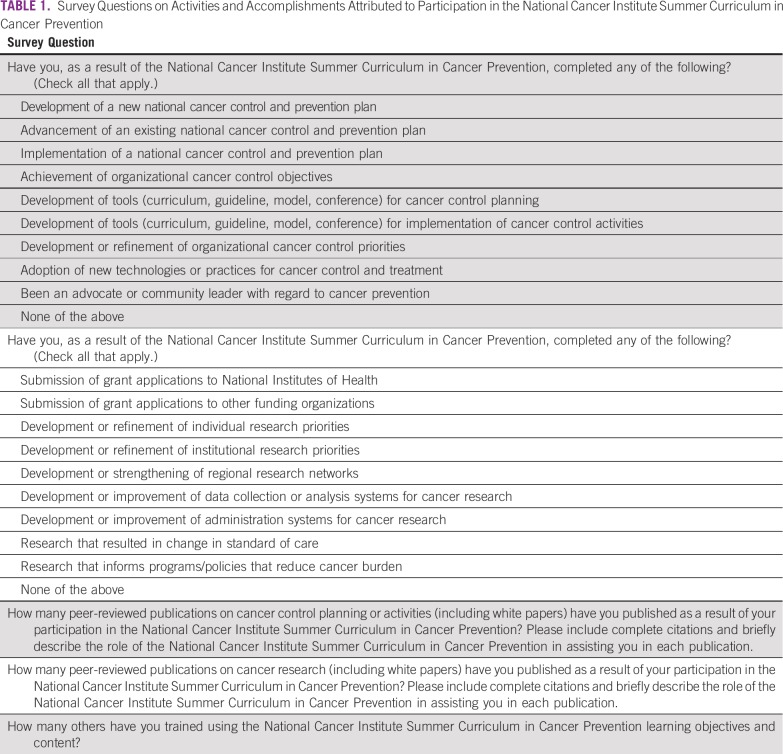
Survey Questions on Activities and Accomplishments Attributed to Participation in the National Cancer Institute Summer Curriculum in Cancer Prevention

The NCI attempted to contact 427 individuals, of whom 357 (83.6%) were successfully contacted. The remaining 70 individuals’ e-mails were undeliverable because of inactive e-mail addresses. Of the 357 individuals whose e-mail invitations were successfully delivered, 156 completed the survey (43.7%). Most undeliverable e-mails were sent to individuals who had participated before 2007 (43 of 70 undeliverable e-mails). Of those individuals who completed the survey, only a handful (n = 16; 10.3%) had participated before 2007. As a result, the analysis was restricted to the 140 respondents who participated between 2007 and 2015.

Given the strong focus of the survey on cancer prevention and control practice on one hand and cancer research on the other, survey respondents were categorized into four subgroups by work sector: (1) academic or research institution, (2) Ministry of Health or policy related institution, (3) health care institution, or (4) nongovernmental organization. Responses were analyzed by subgroup.

SPSS and SAS were used to collate and clean data. Pivot tables in Microsoft Excel were used to calculate descriptive statistics. Excel was also used to facilitate qualitative data analysis. World Health Organization definitions of regional groupings were used to describe country locations of respondents.^11^

## RESULTS

### Respondent Work Sectors and Geographic Locations

Of 140 respondents in the data set, most worked at academic or research institutions (Academic/Research, n = 61), followed by health care institutions (Health Care, n = 41), and Ministry of Health or policy-related institutions (Ministry/Policy, n = 36). Only two respondents worked at nongovernmental organizations. This subgroup was excluded from further analyses because of its small size. The final sample comprised 138 individuals from the other three subgroups.

At the time they participated in the Summer Curriculum, these 138 respondents worked in all six of the World Health Organization regional country groupings. This included 34.1% (n = 47) in the Africa Region, 17.4% (n = 24) in the Southeast Asia Region, 15.2% (n = 21) in the European Region, 14.5% (n = 20) in the Region of the Americas, 11.6% (n = 16) in the Western Pacific Region, and 7.2% (n = 10) in the Eastern Mediterranean Region.

### Summer Curriculum Course Participation

Approximately half of respondents (n = 75; 54.3%) participated in both the Principles course and the Molecular Prevention course. Another 39.1% of respondents (n = 54) participated in the Principles course only, and 6.5% (n = 9) participated in the Molecular Prevention course only. Length of time since participation of respondents averaged 4.5 years, with little variation across subgroups (4.1 years for Academic/Research, 4.7 for Ministry/Policy, and 4.8 for Health Care).

### Continued Work in LMICs

At the time of the survey, almost all respondents (n = 125; 90.6%) worked in the same country as when they participated in the Summer Curriculum. Of the 13 respondents (9.4%) who reported working in a different country than when they participated, five reported staying in the same region and four reported working in another LMIC. Academic/Research respondents were most likely to remain in the same country (95.1%), followed by Health Care (92.7%) and Ministry/Policy (80.6%) respondents.

### Cancer Prevention and Control Practice Activities and Accomplishments Attributed to Participation in the Summer Curriculum

**FIG 1:**
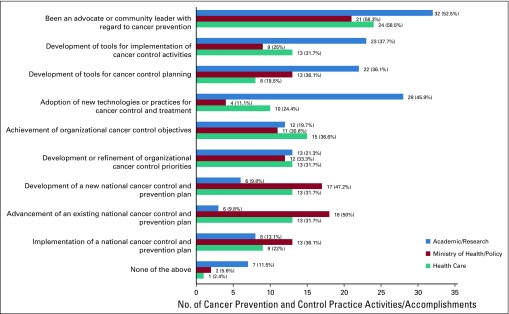
Completed cancer prevention and control practice activities/accomplishments attributed to participation in the National Cancer Institute Summer Curriculum in Cancer Prevention.

**FIG 2:**
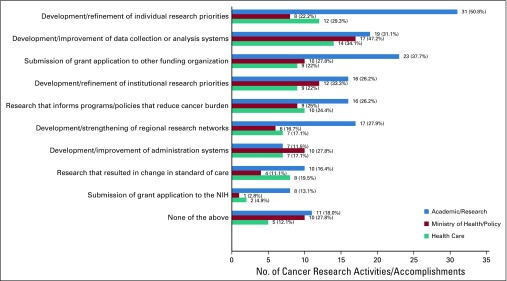
Completed cancer research activities/accomplishments attributed to participation in the National Cancer Institute Summer Curriculum in Cancer Prevention. NIH, National Institutes of Health.

Almost all respondents (94.9%) reported completing at least one cancer prevention and control practice activity/accomplishment or cancer research activity/accomplishment “as a result of” their participation in the Summer Curriculum (Figs 1 and 2). This included 92.8% of respondents who reported completing at least one of nine proposed cancer prevention and control practice activities/accomplishments (Fig 1). Health care respondents were most likely to report one or more of these activities/accomplishments (97.6%), followed by Ministry/Policy respondents (94.4%), and Academic/Research respondents (88.5%).

Across all three subgroups, the most commonly reported completed practice activity/accomplishment attributed to participation in the Summer Curriculum was acting as an advocate or community leader with regard to cancer prevention (Health Care, 58.5%; Ministry/Policy, 58.3%; Academic/Research, 52.5%). The second-most commonly reported practice activity/accomplishment varied by subgroup and was advancement of an existing national cancer control plan (Ministry/Policy, 50.0%), adoption of new technologies or practices for cancer control and treatment (Academic/Research, 45.9%), and achievement of organizational cancer control objectives (Health Care, 36.6%).

### Cancer Research Activities and Accomplishments Attributed to Participation in the Summer Curriculum

Most respondents (81.2%) reported completing one or more of nine proposed cancer research activities/accomplishments as a result of their participation in the Summer Curriculum (Fig 2). Health Care respondents were most likely to report one or more of these activities/accomplishments (87.8%), followed by Academic/Research respondents (81.9%) and Ministry/Policy respondents (72.2%).

The most commonly reported completed cancer research activity/accomplishment attributed to participation in the Summer Curriculum varied by subgroup. Development or refinement of individual research priorities was most common among Academic/Research respondents (50.8%), and development or improvement of data collection or analysis systems for cancer research was most common among respondents in the other two subgroups (Ministry/Policy, 47.2%; Health Care, 34.1%). The second-most commonly reported cancer research activity/accomplishment by subgroup was submission of grant applications to funding organizations other than the National Institutes of Health (Academic/Research, 37.7%), development or refinement of institutional research priorities (Ministry/Policy, 33.3%), and development or refinement of individual research priorities (Health Care, 29.3%).

Analyses found no relationship between number of years since participation and number of reported subsequent cancer prevention and control practice activities/accomplishments or cancer research activities/accomplishments, for all respondents or any subgroup.

### Publications

Just under half of respondents (44.2%) reported publishing “peer-reviewed publications on cancer control planning or activities (including white papers)” or “peer-reviewed publications on cancer research (including white papers),” “as a result of” participation in the Summer Curriculum (Table 2). Health care respondents were most likely to report publications (56.1%), followed by Academic/Research respondents (44.3%) and Ministry/Policy respondents (30.6%).

**TABLE 2:**
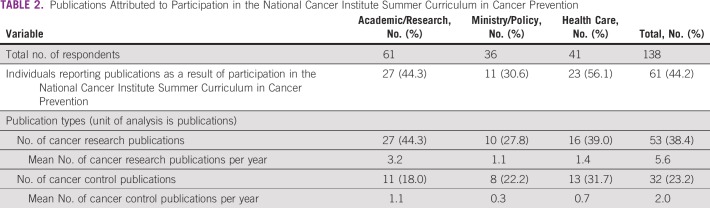
Publications Attributed to Participation in the National Cancer Institute Summer Curriculum in Cancer Prevention

Academic/Research respondents had the highest mean number of publications per year since participation, including both cancer research publications and cancer control publications (3.2 and 1.1 publications per year, respectively), followed by Health Care respondents (1.4 and 0.7 publications per year, respectively), and Ministry/Policy respondents (1.1 and 0.3 publications per year, respectively). Across all three subgroups, there were more cancer research publications than cancer prevention and control publications, per year.

Four key themes emerged from respondents’ text responses to the question of how participation in the Summer Curriculum contributed to their publications. One key theme was that the Summer Curriculum underscored the importance of a public health approach to cancer control and had emphasized, in particular, the importance of prevention. Respondents remarked that they left the course with a stronger understanding of a range of cancer prevention and control approaches and a desire to engage in population-level approaches including surveillance and epidemiology, community-based interventions, communications campaigns, and advocacy. The second theme was that participation in the Summer Curriculum had honed participants’ research interests by educating them about key priorities in cancer research. Respondents mentioned new or refocused research interests in regional variations in cancer prevalence, particular stages along the cancer continuum, and specific research questions. The third theme was that the Summer Curriculum provided content that strengthened respondents’ skills related to research methods and scientific writing. Respondents explained that this assisted them in conducting subsequent research, engaging in subsequent research collaborations, and authoring/coauthoring scientific publications. The fourth theme was that the Summer Curriculum had emphasized the importance of conducting research and disseminating findings via publications. Respondents reported that this increased their commitment to conducting research and publishing their findings.

### Training Others

Most respondents (79.7%) reported using Summer Curriculum learning objectives and content to train at least one person, ranging from 77.8% of Ministry/Policy respondents to 82.9% of Health Care respondents (Table 3). Respondents reported having had a range of 0 to 1,000 trainees, with a marked right-tailed distribution. Most respondents (65.2%) trained 1 to 50 individuals, and few respondents trained more than 200 individuals (5.1%). Accounting for years since participation in the Summer Curriculum, Academic/Research and Health Care respondents reported a median of two individuals trained per year, and Ministry/Policy respondents reported a median of one individual trained per year.

**TABLE 3:**
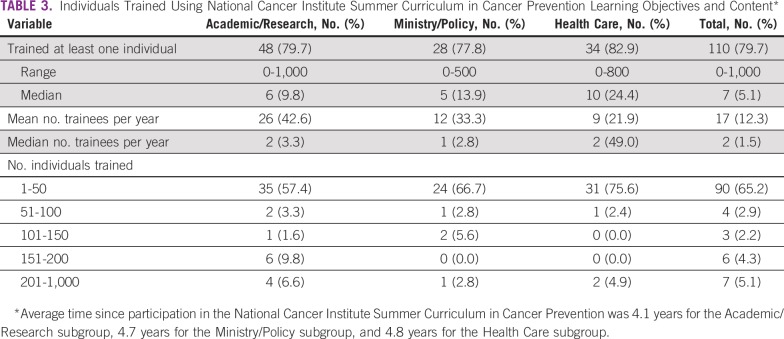
Individuals Trained Using National Cancer Institute Summer Curriculum in Cancer Prevention Learning Objectives and Content*

## DISCUSSION

The growing burden of cancer in LMICs has created an imperative to develop local capacity for cancer prevention and control through a range of approaches. The NCI Summer Curriculum provides an opportunity for LMIC-based professionals to obtain rigorous training in cancer prevention and control via a short-term program.

This survey provides evidence that Summer Curriculum alumni who were based in LMICs when they participated continued to work in cancer prevention and control in LMICs. Almost all respondents reported at least one subsequent cancer prevention and control practice or cancer research activity/accomplishment (94.9%). In addition, almost all respondents reported working in the same LMIC as when they participated in the program (90.6%).

A common concern regarding trainings hosted in HIC settings for highly trained LMIC researchers is that they may draw participants away from LMICs, where their expertise is greatly needed.^12^ Our findings suggest that this is not the case for this short-term training program and that it in fact contributes to capacity building in LMICs, both through the training of Summer Curriculum participants and those they subsequently train in LMICs using Summer Curriculum objectives and content.

The survey also provides a window into the cancer prevention and control activities/accomplishments that Summer Curriculum alumni working in LMICs found were most assisted by their participation in the program. The variation by subgroup in the most commonly reported cancer control practice and cancer research activities/accomplishments attributed to participation in the Summer Curriculum reflects the variety of activities that respondents engaged in (eg, advocacy, national cancer control planning, and grantsmanship) and the variety of activities for which the Summer Curriculum was helpful. These findings may help to inform planning for the topics and skills addressed in future cancer prevention and control training programs for LMIC-based participants.

Just under half of survey respondents (44.3%) credited the Summer Curriculum with contributing to their authorship of peer-reviewed publications on cancer control or cancer research. The four key ways that respondents reported the Summer Curriculum informed their work on these publications help to shed light on the strengths of the Summer Curriculum. These themes also help to explain respondents’ reported activities/accomplishments in cancer prevention and control practice and research completed as a result of participation in the Summer Curriculum.

Most respondents (79.7%) reported using Summer Curriculum learning objectives and content to train others. This points to the potential of a train-the-trainer model to disseminate training in cancer prevention and control in LMICs. The skewed distribution of number of trainees per respondent suggests that training occurred mainly in individual and small group contexts, particularly in the Health Care subgroup, whereas some respondents who were primarily in the Academic/Research subgroup provided training in large group settings. These findings point to the need for training that includes teaching strategies for both small- and large-group formats and for audiences with varied focus areas.

The proliferation of online training in recent years points to another potentially fruitful approach. Project ECHO (Extension for Community Healthcare Outcomes) is one example of a Web-based platform for peer-based training and learning that is currently being used by the NCI for cancer control and prevention training among LMIC-based participants.^13,14^ All of these training approaches (train-the-trainer, online, and peer based) have the potential to more rapidly advance capacity in cancer prevention and control in LMICs.

A limitation of this survey was the number of alumni whose e-mail invitations were undeliverable. This could have influenced our findings because those who were not reachable may have been more likely to have changed their place of work, and therefore their professional activities, than those who were reached. A second limitation was the wording of certain survey questions. Specifically, the two core scales about cancer prevention and control and cancer research activities/accomplishments asked about those that were “completed.” Because some of these activities/accomplishments may have been in progress at the time of the survey, the question wording may have resulted in under-reporting. A third limitation was reliance on self-report. Although this approach was appropriate to the resources and time available for this evaluation, it may have had an impact on reporting.

In conclusion, strengthening LMIC-based expertise in cancer prevention and control is an essential component of capacity building to address the growing burden of cancer in LMICs. The NCI Summer Curriculum provides an opportunity for LMIC-based professionals to obtain rigorous training via a short-term program. This survey sheds light on the professional activities that LMIC-based alumni of the Summer Curriculum found were most assisted by their participation and provides insights into how the Summer Curriculum informed these activities. It also highlights how these participants are training others in LMICs using content from the Summer Curriculum. Findings suggest future directions for this and other training opportunities in cancer prevention and control for LMIC-based participants, including train-the-trainer models, online learning, and peer-based learning approaches.
